# T2 mapping in different cardiomyopathies: first clinical experience

**DOI:** 10.1186/1532-429X-15-S1-P53

**Published:** 2013-01-30

**Authors:** Florian Bönner, Mirja Neizel, Sebastian Gruenig, Christoph Jacoby, Malte Kelm, Burkhard Sievers

**Affiliations:** 1 Department of Cardiology, Pneumology and Angiology, Heinrich Heine University Düsseldorf, Düsseldorf, Germany; 2 Department of Molecular Cardiology, Heinrich Heine University, Düsseldorf, Germany

## Background

Structural myocardial changes accompany myocardial pathologies such as myocardial ischemia, myocarditis, hypertrophy (HCM) and myocardial remodeling. These changes affect T2 relaxation times which can non-invasively be detected by cardiovascular magnetic resonance imaging (CMR). Since the interpretation of T2 weighted images remains a "risky business" due to subjectivity, the purpose of our study was to evaluate direct T2 value quantification by T2 mapping in different types of cardiomyopathies.

## Methods

T2 maps were calculated from images recorded with a gated multislice GRASE sequence (9 echos, separated by TE = 7 ms, TR = 750 ms, TA = 5 min, Voxel Size: 2x2x10mm, fat saturation). For creation of T2 maps an exponential decay curve was fitted to the intensity progression of each pixel within the images obtained from the multi echo sequence using a dedicated software based on the graphical programming language LabVIEW (National Instruments, Austin, TX). T2 value distribution in 3 short axis slices (apical, mid-ventricular and basal) were evaluated. Mean, median and standard deviation of values were calculated automatically after manual identification of left ventricular myocardium. T2 maps were analysed in patients with low grade (14-50ng/l) high sensitive troponin T (hsTnT) elevation (n=10), myocarditis (n=10), HCM (n=10) and in a young (mean age: 25 years; n=5) as well as elderly subjects (mean age: 64 years; n=10) without known structural heart disease (control group).

## Results

**Figure 1:**
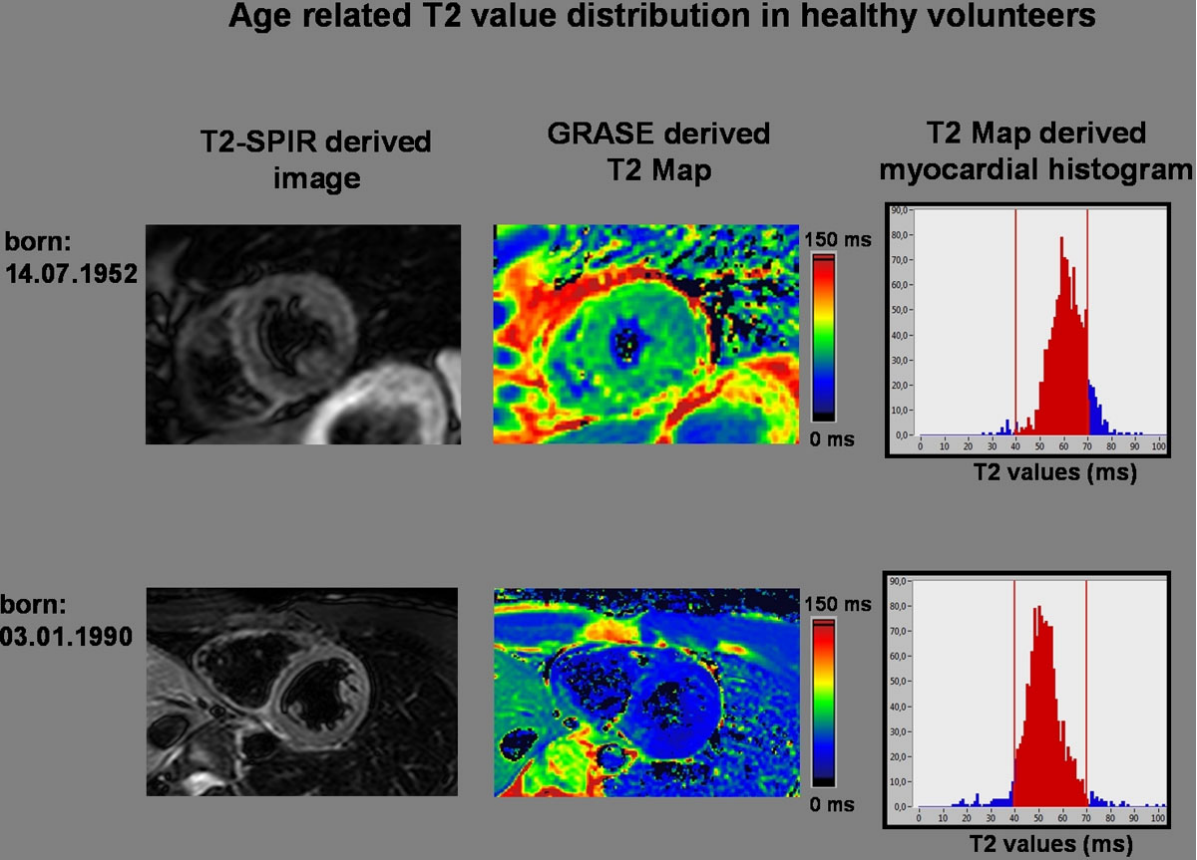


GRASE was successfully implemented into clinical routine protocols (+ Cine-MRI, LGE and T2-SPIR) and resulted in a mean prolongation of the imaging protocol of only 5 minutes. Due to non gaussian distributed T2 values, median T2 values were analysed (Figure 1). In case of the age matched control group, median T2 value was 58.9±4.5ms (Figure 1). The analysis revealed that median T2 values (apical: 60.3±4.2ms, midventricular: 59.4±4.7ms, basal: 58.7±4.5ms) drop significantly from apex to basis in each individual. Median T2 values were only 1.8ms different from mean T2 values. Younger age was related with decreased T2 values (Figure 1: median T2 value: 48.7±3.7ms). Patients with HCM displayed significant greater median T2 values (67ms±6.2ms, p<0.05) than the age matched control group. T2 mapping identified focal lesions (92ms±24.2ms) caused by low grade ischemia, hsTnT release and/or epicardial myocarditis without any contrast in conventional T2 SPIR and LGE.

## Conclusions

In summary, T2 mapping could be successfully implemented in our clinical routine protocols. We found that (i) there seems to be an age related increase in T2 values in the absence of known cardiac disease and (ii) our sequence is able to identify regions with highly increased T2 values caused by myocarditis or low grade ischemia without any contrast in conventional T2 SPIR or LGE.

## Funding

none

